# Notch1 and 4 Signaling Responds to an Increasing Vascular Wall Shear Stress in a Rat Model of Arteriovenous Malformations

**DOI:** 10.1155/2014/368082

**Published:** 2014-01-20

**Authors:** Jian Tu, Yang Li, Zhiqiang Hu

**Affiliations:** ^1^Australian School of Advanced Medicine, Macquarie University, 2 Technology Place, North Ryde, Sydney, NSW 2109, Australia; ^2^Department of Neurosurgery, The 9th Medical Clinical College of Beijing University, Beijing 100038, China

## Abstract

Notch signaling is suggested to promote the development and maintenance of cerebral arteriovenous malformations (AVMs), and an increasing wall shear stress (WSS) contributes to AVM rupture. Little is known about whether WSS impacts Notch signaling, which is important for understanding the angiogenesis of AVMs. WSS was measured in arteriovenous fistulas (AVF) surgically created in 96 rats at different time points over a period of 84 days. The expression of Notch receptors 1 and 4 and their ligands, Delta1 and 4, Jagged1, and Notch downstream gene target Hes1 was quantified in “nidus” vessels. The interaction events between Notch receptors and their ligands were quantified using proximity ligation assay. There was a positive correlation between WSS and time (*r* = 0.97; *P* < 0.001). The expression of Notch receptors and their ligands was upregulated following AVF formation. There was a positive correlation between time and the number of interactions between Notch receptors and their ligands aftre AVF formation (*r* = 0.62, *P* < 0.05) and a positive correlation between WSS and the number of interactions between Notch receptors and their ligands (*r* = 0.87, *P* < 0.005). In conclusion, an increasing WSS may contribute to the angiogenesis of AVMs by activation of Notch signaling.

## 1. Introduction

Cerebral arteriovenous malformations (AVMs) consist of an abnormal tangle of fistulas that shunt blood from the arterial system to the venous system without an intervening capillary bed [1]. As the direct communication of high pressure arterial blood into the thin-walled venous vessels, AVMs present an altered hemodynamic state [2]. The increased shear stress upon the vessel wall in combination with the structural immaturity of AVM vasculature presents an increased risk of rupture. Their effects on the blood flow of the surrounding parenchyma may be responsible for other clinical manifestations of AVMs. It has been suggested that high flow conditions and the increased diameter and variability of the vessels within the nidus predispose to the development of turbulent flow [3]. While the existence of turbulence has not been demonstrated by direct measurement *in vivo *[4], many of the physiological and histological hallmarks of turbulent flow are present within AVMs. The increased rate of endothelial cell turnover [5], focal dilatation of vessels [3], and platelet aggregation [6] are indicative of high wall shear stress (WSS) associated with turbulence. It is unclear whether these derangements represent a primary abnormality of the AVM blood vessels or whether they are a secondary response to the abnormal hemodynamic environment within the AVM. The altered expression of angiogenic factors may be important in the vascular remodeling and continued angiogenesis that occur in these dynamic lesions [7]. Until the precise mechanism of action of many of these angiogenic factors is clarified, it is difficult to draw conclusions on the relevance of these abnormalities to AVM pathogenesis.

Notch signaling pathway has been implicated as a regulator of vascular angiogenesis and in the development of the human AVMs [8, 9]. Activation of the Notch receptor1 or 4 in mice causes AVMs-like abnormalities [8, 10–12]. Recently, normalization of Notch4 has been suggested as a strategy to reduce blood vessel size in a mouse model of AVMs [13]. Notch1 is expressed in vascular endothelial cells and smooth muscle cells while Notch4 is expressed primarily in endothelial cells [14]. Notch ligands, Delta1 and 4, and Jagged1 are expressed in vascular endothelial cells and smooth muscle cells [15, 16]. Both Notch receptors and their ligands are transmembrane proteins; therefore, signaling is restricted to neighboring cells. Although the intracellular transduction of the Notch signal is remarkably simple, with no secondary messengers, this pathway functions in an enormous diversity of developmental processes. The specific roles of individual Notch receptors and their ligands in human vascular homeostasis are little known. The majority of knowledge implicating the Notch signaling in vessel homeostasis and development has arisen through gain and loss of function studies in mice [17, 18]. Observations in mice suggest that Notch1 plays a role in angiogenesis [18] and AVM pathogenesis [8]. Notch4 is involved in the initiation and maintenance of arteriovenous communications [12]; though Notch4 is not critical to vascular development, it shares functional redundancy with Notch1 in vascular development [17]. Delta1 is suggested to be critical to vascular maturation and vessel integrity [19]. Delta4 plays a critical role in early vascular remodelling, arterial and venous specialization, and Notch1-mediated signaling in early vascular development [20, 21]. Jagged1 contributes to vascular maturation and plays a distinct role in Notch1 signaling [22, 23]. The direct targets of Notch signaling remain vague. Notch expression activates transcription of Hairy/Enhancer of Split (Hes) family genes and subsequently results in repression of Hes target genes [24], many of which are tissue-specific transcriptional activators [25]. Thus, Notch activation of Hes can modulate cellular differentiation. It has been reported that Notch signaling pathway responds to Notch1 activator by increased angiogenesis and Jagged1 inhibitor by reduced angiogenesis in adult rats [9]. However, the knowledge of how does Notch signaling pathway respond to an increasing WSS in AVM vessels remains absent from the literature. This study was undertaken to examine whether endothelial Notch signaling responds to an increasing WSS in the “nidus” vessels in a rat model of AVMs. If so, how does WSS regulate the function of Notch signaling pathways?

## 2. Materials and Methods

### 2.1. AVF Rat Model

**Figure 1:**
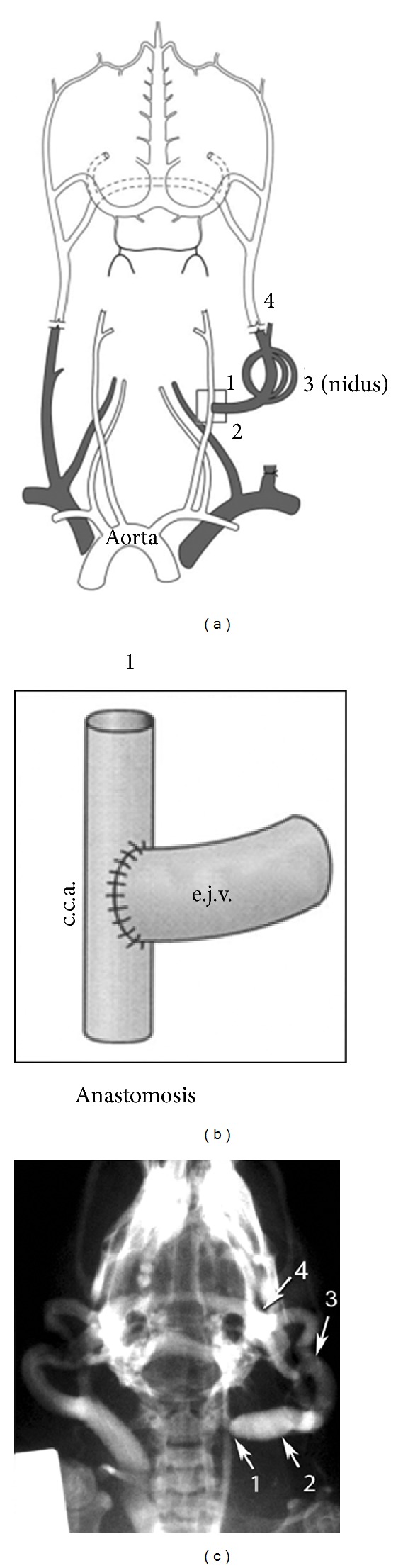
Arteriovenous fistula in a rat model of arteriovenous malformation. (a) Schematic representation shows an AVF in a rat model of AVM. The normal primary outflow for intracranial venous blood is the external jugular vein (e.j.v.) via the posterior facial vein and the vein from transverse sinus. The left external jugular vein is ligated at the confluence of subclavian vein, and (b) an end-to-side anastomosis was performed onto the left common carotid artery (c.c.a.). 1: carotid-jugular anastomosis; 2: arterialized feeding vein; 3: “nidus” consists of dilated small vessels and capillaries; 4: draining vein. (c) Representative angiogram obtained 42 days after creation of the rat AVF model. Portions of the rat AVF model are indicated: 1: proximal fistula; 2: arterialized jugular vein; 3: “nidus”; 4: draining vein.

Animal experimentation was approved (Animal Ethics Approval numbers 08/97a, 2009/047, 2009/048, and 2010/037) and performed in accordance with the guidelines of the institutional Experimental Animal Care and Ethics Committee, Guide for the Care and Use of Laboratory Animals (Institute for Laboratory Animal Research, National Research Council, Washington, DC: National Academy Press, 1996), and the Code of Practice for the Care and Use of Animals for Scientific Purposes [26]. AVF rat model was created in 96 Sprague-Dawley male rats (7 weeks old, 230 ± 9 g) by an anastomosis of the left common carotid artery to the left external jugular vein as shown in Figure 1 and as previously reported [27–31]. Rats were allowed to acclimatize to new surroundings before the experiment began. Surgical procedures were not performed in the presence of other rats. General anaesthesia was induced using a mixture of 4% isoflurane and oxygen (2 L/min) via a nose cone. The depth of anaesthesia was assessed using the respiratory rate and by checking the hind limb withdrawal to pain. No procedure was commenced until there was a consistent absence of response to pain. A heating blanket was used for the duration of the procedure.

The procedure was performed in a sterile field using aseptic technique. The left common carotid artery (CCA) was exposed, and blood flow was measured through the CCA using a 1 mm Doppler ultrasonic probe (Transonic Systems, Ithaca, NY, USA). The left external jugular vein (EJV) was then exposed and ligated with 10/0 nylon suture at its junction with the subclavian vein. An aneurysm clip was placed across the rostral EJV. Microclips were also applied proximally and distally on the CCA, and a small arteriotomy made on the lateral aspect. An end-to-side anastomosis of the EJV to the CCA was performed using a continuous 10/0 nylon suture. The clips were sequentially removed from the EJV, distal CCA, and proximal CCA. Blood flow was measured through the proximal CCA and the vein using 1 and 2 mm Doppler ultrasonic probes (Transonic Systems). The wound was closed, and isoflurane was turned off, allowing the animal to breathe oxygen until the time of awakening. Once awake, the animal was placed in an individual cage and housed singly for one week postoperatively. Observations were carried out daily for the first week and then weekly thereafter. Observations included weight, assessment of motor function, behavior, and wound health. Six weeks after surgery, angiography was performed in 6 AVF rats, and their dilated small vessels and capillaries formed a “nidus” (Figure 1). There was no evidence of significant morbidity and mortality associated with AVF formation. The sham-AVF controls were treated identically but did not receive AVF formation surgical procedures. The sham-AVF controls were subjected to the same analysis as AVF rats. Data obtained from sham-AVF controls were expressed as the pre-AVF formation at −1 day time point in comparison with AVF rats at different time points over a follow-up period of 84 days after AVF formation.

### 2.2. Vascular WSS in AVF Rat Model

Blood blow was measured in the carotid artery before and after fistula creation, and in the jugular vein after fistula creation, using a flow probe (1 or 2 RB, Transonic Systems) connected to a transit time perivascular flowmeter (T420, Transonic Systems) [27]. Blood flow rate was recorded through a data acquisition system (PowerLab/8sp System, ADInstruments, Castle Hill, NSW, Australia). Shear stress was estimated using the Poiseuille formula *τ* = 4*ηQ*/(*πR*_*i*_^3^), where *τ* is wall shear stress, *η* is blood viscosity, *Q* is blood flow rate, and *R*_*i*_ is the internal radius. It has been demonstrated that Poiseuille's law can be applied to the flow within blood vessels of diameters greater than 0.1 mm [32]. Therefore, it is applicable to this arteriovenous fistula model.

### 2.3. Immunohistochemistry

Rats were anaesthetized and perfused with 4% paraformaldehyde. Specimens, including carotid-jugular anastomosis, arterialized feeding vein, the “nidus,” and draining vein, were embedded in tissue freezing medium (ProSciTech, QLD, Australia) with liquid nitrogen for immunohistochemistry and proximity ligation assay. Immunohistochemistry was performed in specimens obtained from 32 AVF rats and 4 sham AVF controls as previously described [28–31, 33]. Briefly, sections were washed, and nonspecific binding was blocked by 10% horse serum. Anti-rat primary antibody (Table 1) was applied and incubated at 4°C overnight. Slides were washed, incubated with Alexa Fluor conjugated secondary antibody (Table 1) for 2 hours in dark, and examined using a confocal microscope (Leica SP5, Germany), and imaging data was analyzed using Leica LAS AF software. Each staining was triplicated and repeated 6 times. Fluorescence intensity units (FIU) were corrected using primary antibody controls. The FIU has been quantified as mean gray value. Slides were viewed by three observers blinded to the sample nature.

### 2.4. Proximity Ligation Assay

Proximity ligation assay (PLA) was applied to examine the interaction between Notch receptor and its ligand in specimens obtained from 64 AVF rats and 8 sham AVF controls as previously described [34]. All reagents used for the PLA were purchased from Olink Bioscience (Uppsala, Sweden). The *in situ* PLA was performed according to the manufacturer's instructions. Briefly, tissue sections were permeabilized in 0.2% TX-100, 0.5% BSA in PBS, then blocked in 10% BSA in PBS, and incubated with anti-rat primary antibodies (Table 1). Duolink MINUS and PLUS conjugated secondary antibody incubation, ligation, and amplification steps for PLA were performed as suggested by Olink using 40 *μ*L volume. Following amplification, slides were washed for 10 min in Olink Buffer B, pH 7.5, followed by a 10 min wash in 0.5% BSA. Fluorescent images were obtained using a confocal microscope (Leica TCS SP5X, Wetzlar, Germany). Z-Stacks were composed of 6 consecutive images with a total Z volume of 12 *μ*m.

Images captured for PLA events were analyzed using Leica LAS AF software (Version 2.4.1; Leica Microsystems GmbH, Wetzlar, Germany). First, Z-stack images were converted into maximum representations. Three polygon regions of interest were drawn evenly along the vessel lumen 5 *μ*m into the tunica intima as to envelope the vessel's endothelium. Three more circular regions of interest were evenly placed within the tunica media. The regions of interest were between 0.5 and 1.0 mm^2^ in size. The positive PLA events were observed as fluorescent particles (size from 2 to 50 pixels in diameter). When PLA events merged to create particles larger than 50 pixels, the area was measured, and the number of events was assumed to be particle area divided by 10 since 10 pixels were the median size of most PLA events. The number of fluorescent spots obtained from PLA in regions of interest were automatically quantified and recorded. A threshold of 100 (gray values) was set for a positive signal prior to signal counting. To account for nonspecific signals, “background” signal/mm^2^ values for each specimen's endothelial and medial regions of interest were generated from each specimen's negative control and then subtracted from each respective region of interest signal/mm^2^ value. The number of PLA events was assessed by two observers blinded to the sample nature.

### 2.5. Data Analysis

Data were expressed as means ± SE (number of experiments). Statistical difference between groups was determined using the unpaired two-tailed *t*-test. When there were more than two groups, differences were analyzed using analysis of variance if the variances were equal, and the Mann-Whitney nonparametric test if variances were unequal [35]. Linear regressions were calculated using a statistical computer package, Number Cruncher Statistical Systems [35]. A value of *P* < 0.05 was considered statistically significant.

## 3. Results

### 3.1. Hemodynamic Changes in an AVF Rat Model

**Figure 2:**
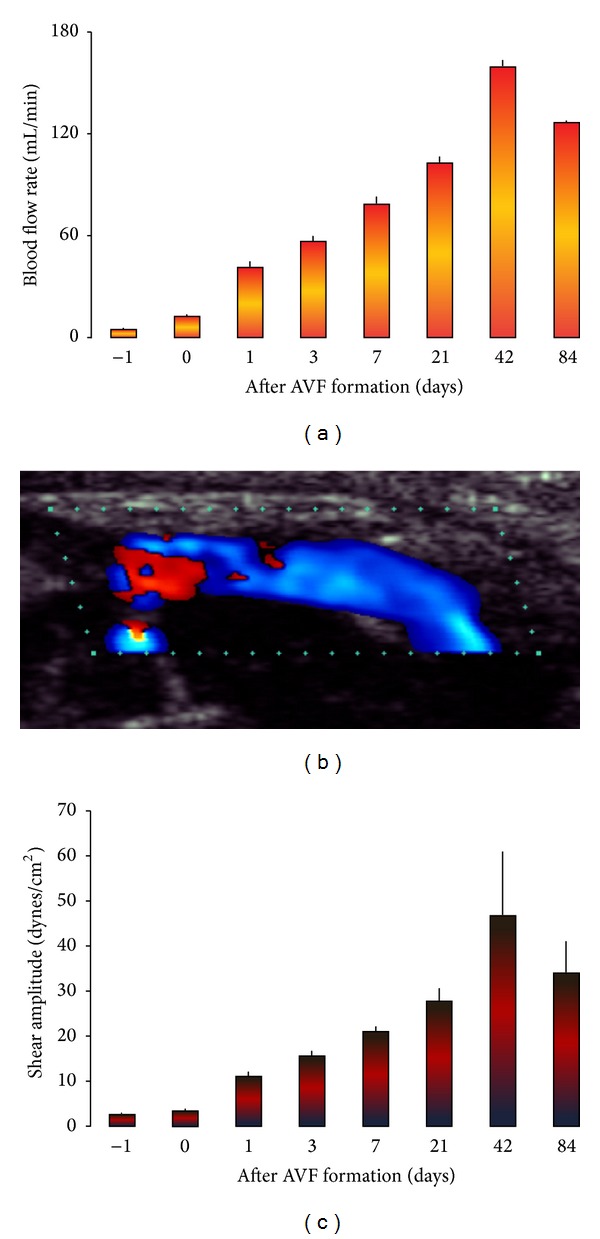
Pulsatile blood flow and shear stress in the arterialized jugular vein. (a) Fistula blood flow rate. (b) Representative Doppler ultrasound image of blood flow through the arterialized jugular vein. Red and blue colors indicate two different blood flow directions, suggesting turbulent blood flow at the proximal fistula. Blue color indicates the same blood flow direction, suggesting laminar blood flow at the through the arterialized jugular vein. (c) Fistula shear stress. Data were expressed as means ± SE (*n* = 6). Day −1: flow in the common carotid artery prior to fistula creation.

Before anastomosing the caudal end of the external jugular vein to the side of the common carotid artery, the blood flow through the left carotid artery was 5.2 ± 0.07 mL/min. Immediately after fistula creation, the common carotid flow (proximal to the fistula) increased by 140 ± 7% (*P* < 0.05). The blood flow changes over 84 days are depicted in Figure 2. Flow rate increased with time and peaked at 42 days (Figure 2(a)). There was a strong positive correlation between fistula blood flow and time up to 42 days after fistula formation (*r* = 0.96; *P* < 0.001). There was no statistical difference in the flow rate measured between day 42 and day 84. The maximum flow at day 42 was 11 times greater than the initial fistula flow.

Flow through the fistula was pulsatile. Turbulent blood flow was observed at the proximal fistula as red and blue colors in Figure 2(b) indicating two different blood flow directions. Laminar blood flow was observed at the through the arterialized jugular vein as blue color in Figure 2(b) indicates the same blood flow direction. There was a net positive mean shear stress that increased over time (Figure 2(c)). Shear amplitude in the fistula vein increased from 3.5 to 46 dynes/cm^2^ from day 0 to day 42. There was a strong positive correlation between shear stress and time up to 42 days (*r* = 0.97; *P* < 0.001). There was no statistical difference in shear stress between day 42 and day 84. The maximum shear amplitude achieved at day 42 was 14 times the level at the time of fistula formation.

### 3.2. Increasing WSS Induces Apoptosis in an AVF Rat Model

**Figure 3:**
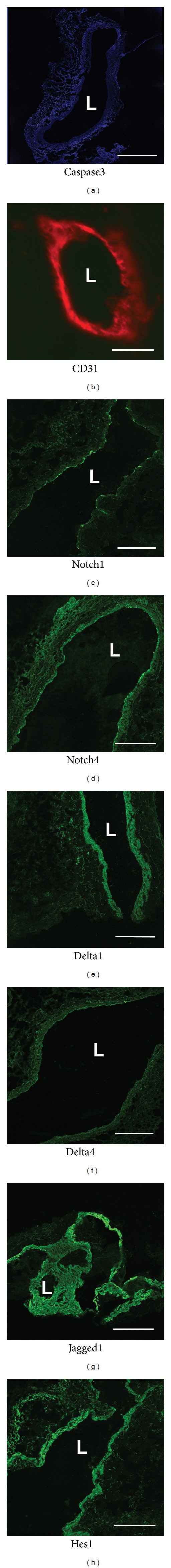
The positive immunofluorescence of caspase3 staining in blue in the “nidus” vessels. CD31 was stained positively in red. The positive immunofluorescence of Notch1 and 4 receptors, their ligands Jagged1, Delta1 and 4, and Notch downstream target Hes1 in green in the “nidus” vessels 42 days after AVF formation. L: lumen. Immunohistochemistry, bar = 300 *μ*m.

**Figure 4:**
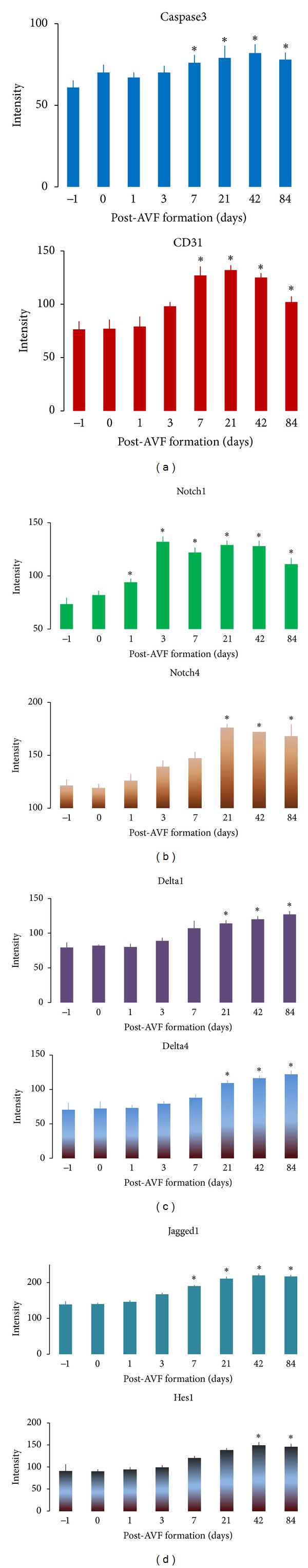
The intensity of immunofluorescence of caspase3, CD31 (a), Notch1 and 4 receptors (b), their ligands Delta1 and 4 (c), Jagged1 and Notch downstream target Hes1 (d) in the “nidus” vessels was quantified using a confocal microscope over a period of 84-day after AVF formation. Data were expressed as means ± SE of 4 rats at each time point. *P < 0.05 paired comparison between pre- (−1 day) and post-AVF formation at different time points. There was a positive correlation between the intensity of immunofluorescence of caspase3 and time (*r* = 0.8866, *P* < 0.005), Notch4 and time (*r* = 0.7252, *P* < 0.05), Delta1 and time (*r* = 0.8489, *P* < 0.008), Delta4 and time (*r* = 0.8874, *P* < 0.004), and Hes1 and time (*r* = 0.8185, *P* < 0.02) over a period of 84 days after AVF formation.

Caspase3 was selected as a marker for apoptosis. The levels of caspase3 expression in “nidus” vessel wall in the AVF rats over a period of 84 days after AVF formation were shown in Figures 3(a) and 4(a). There was a significant upregulation of caspase3 expression in “nidus” vessels after AVF formation. The levels of caspase3 overexpression were 10% at 1 day after AVF formation and peaked at 35% at 42 days after AVF creation (*P* < 0.05).

### 3.3. Expression of Notch Receptors and Their Ligands in an AVF Rat Model

CD31 was selected as a marker for endothelium of the “nidus” vessel wall. Its expression was shown in Figures 3 and 4. The expression of Notch receptor1, Notch receptor4, Delta-like1, Delta-like4, Jagged1, and Hes1 was predominantly expressed in the endothelium of the “nidus” vessel wall (Figure 3).

### 3.4. Increasing WSS Activates Notch Receptors 1 and 4

The expression of Notch receptor1 was significantly upregulated in the “nidus” vessel wall since day 1 after AVF formation (Figure 4(b)). The level of Notch1 expression increased by 81% on day 3 (*P* < 0.01), which was sustainable over an 84-day follow-up period. Prior to AVF formation, the level of Notch receptor4 expression was greater than that of Notch1 expression (*P* < 0.01; Figure 4(b)). There was a 3-week delay in upregulated Notch4 expression comparing with that of Notch1 (Figure 4(b)). The expression of Notch4 was significantly upregulated by 45% on day 21 after AVF formation (*P* < 0.01; Figure 4(b)) and sustained for another 9 weeks of the follow-up period. There was a positive correlation between the levels of Notch4 expression and time over a period of 84 days after AVF formation (*r* = 0.7252, *P* < 0.05).

### 3.5. Increasing WSS Activates Notch Receptor Ligands and Hairy Enhancer of Split1

The expression of Notch receptor ligands, Delta1 and 4, was significantly upregulated in the “nidus” vessel wall 3 weeks after AVF formation (*P* < 0.05; Figure 4(c)) while Jagged1 was responsively upregulated 2 weeks earlier than that of Delta1 and 4 (*P* < 0.04; Figure 4(d)). The highest levels of Delta1 and 4 expression were observed on day 84 following AVF formation, which increased by 61% and 74%, respectively, compared to the pre-AVF formation (*P* < 0.01). The greatest level of Jagged1 expression was observed on day 42 after AVF formation, which elevated by 58% compared to the pre-AVF formation (*P* < 0.01). Increasing expression of Delta1 and 4 and Jagged1 was positively correlated with time following AVF formation (*r* = 0.8489, *P* < 0.008; *r* = 0.8874, *P* < 0.004; *r* = 0.7734, *P* < 0.03; resp.).

Hes1 represents the overall activity of Notch signaling. The level of Hes1 expression peaked in the “nidus” vessel wall 42 days after AVF formation, which was 64% greater than the pre-AVF formation (*P* < 0.05). There was a positive correlation between Hes1 expression and time following AVF formation (*r* = 0.8185, *P* < 0.02). This phenomenon was also observed in the expression time-course of Jagged1 (Figure 4).

### 3.6. Increasing WSS Activates Interaction between Notch Receptors and Their Ligands

**Figure 5:**
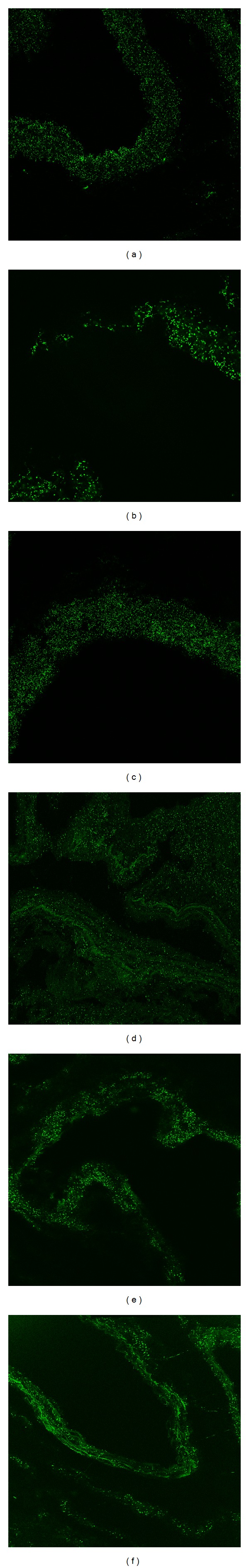
Blood flow shear stress induces the interaction events between Notch receptor1 or 4 and their ligands in the “nidus” vessel wall 42 days after AVF formation. Green dots indicate interaction events: the interaction events between Notch receptor1 and its ligand Delta1 (a), Notch1 and Delta4 (b), Notch1 and Jagged1 (c), Notch receptor4 and Delta1 (d), Notch4 and Delta4 (e), and Notch4 and Jagged1 (f), respectively. *In situ* proximity ligation assay, original magnification ×10 for (a), and ((c)–(f)); ×63 for (b).

**Figure 6:**
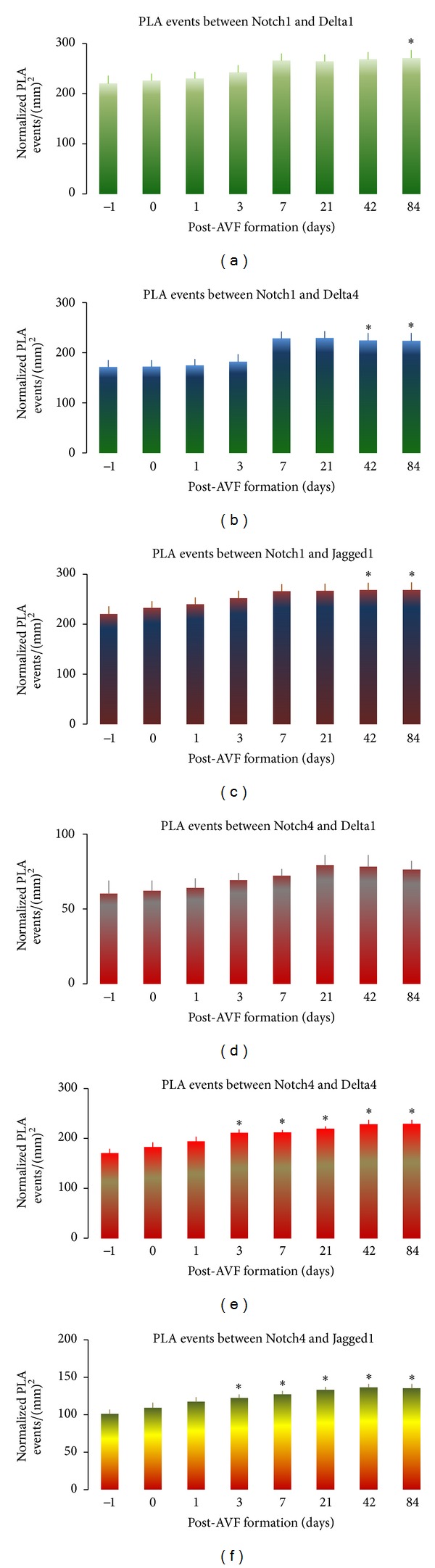
Blood flow shear stress increases interaction events between Notch receptor1 or 4 and their ligands in the “nidus” vessel wall with time over a period of 84 days after AVF formation. Time-course of interaction events between Notch receptor1 and its ligand Delta1 ((a); *r* = 0.716, *P* < 0.05), Notch1 and Delta4 ((b); *r* = 0.6289, *P* < 0.05), Notch1 and Jagged1 ((c); *r* = 0.635, *P* < 0.05), Notch receptor4 and Delta1 ((d); *r* = 0.6727, *P* < 0.05), Notch4 and Delta4 ((e); *r* = 0.7258, *P* < 0.05), and Notch4 and Jagged1 ((f); *r* = 0.6937, *P* < 0.05), respectively. Data were expressed as means ± SE of 4 rats at each time point. *P < 0.05 paired comparison between pre- (−1 day) and post-AVF formation at different time points.

Confirmation of interaction events between Notch receptor1 or 4 and Delta1, Delta4, or Jagged1 in the “nidus” vessel wall was performed using *in situ* proximity ligation assay (Figure 5). Proximity ligation revealed that the number of interaction events between Notch receptor1 and Delta1 in the “nidus” vessel wall was significantly upregulated 84 days after AVF formation, which was 23% more than the pre-AVF formation (*P* < 0.05; Figure 6(a)). The number of interaction events between Notch receptor1 and Delta1 was positively correlated with time following AVF formation (*r* = 0.716, *P* < 0.05). The number of interaction events between Notch1 and Delta4 or Notch1 and Jagged1 in the “nidus” vessel wall was significantly upregulated 42 and 84 days after AVF formation, which was 31% and 22% more than the pre-AVF formation, respectively (*P* < 0.05; Figures 6(b) and 6(c)). There was a positive correlation between the number of interaction events between Notch1 and Delta4 or Notch1 and Jagged1 and time following AVF formation (*r* = 0.6289, *P* < 0.05; *r* = 0.635, *P* < 0.05; resp.).

The number of interaction events between Notch4 and Delta1 in the “nidus” vessel wall was positively correlated with time after AVF formation (*r* = 0.6727, *P* < 0.05; Figure 6(d)). The number of interaction events between Notch4 and Delta4 in the “nidus” vessel wall was significantly upregulated 3 days after AVF formation, which ranged from 24% to 35% over a period of day−3 to −84 (*P* < 0.05; Figure 6(e)). The number of interaction events between Notch4 and Delta4 was positively correlated with time post-AVF formation (*r* = 0.7285, *P* < 0.05). The number of interaction events between Notch4 and Jagged1 in the “nidus” vessel wall was significantly upregulated 3 days after AVF formation, which ranged from 21% to 35% over a period of day−3 to −84 (*P* < 0.05; Figure 6(f)). The number of interaction events between Notch4 and Jagged1 was positively correlated with time after AVF formation (*r* = 0.6937, *P* < 0.05).

### 3.7. A Positive Correlation between WSS and the Number of Interaction Events between Notch Receptors and Their Ligands

**Figure 7:**
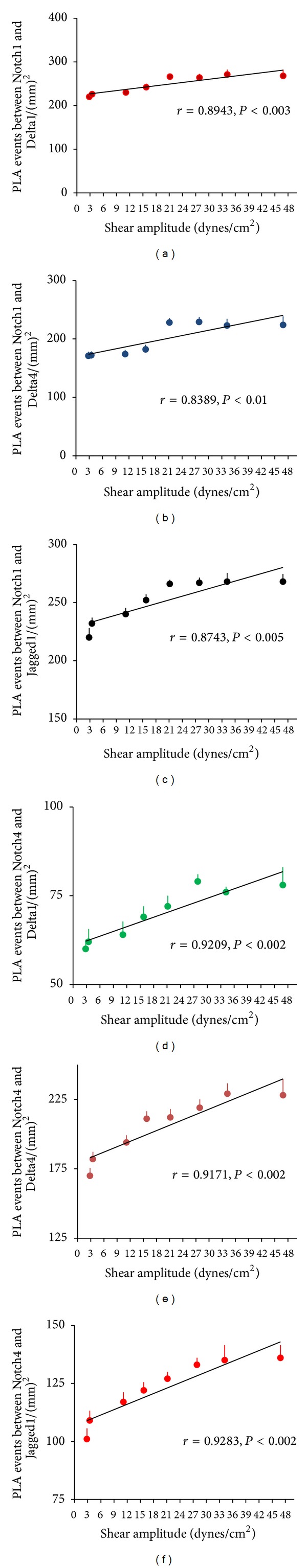
A positive correlation between WSS and the interaction events between Notch receptor1 or 4 and their ligands in the “nidus” vessel wall over a period of 84 days after AVF formation. A positive correlation between WSS and the interaction events between Notch1 and Delta1 (a), Notch1 and Delta4 (b), Notch1 and Jagged1 (c), Notch4 and Delta1 (d), Notch4 and Delta4 (e), and Notch4 and Jagged1 (f), respectively. Data were expressed as means ± SE of 4 rats at each time point. *P < 0.05 paired comparison between pre- (−1 day) and post-AVF formation at different time points.

A positive correlation was observed between an increasing WSS and the interaction events between Notch receptor 1 or 4 and their ligands in the “nidus” vessel wall over a period of 84 days following AVF formation (Figure 7). An increasing WSS positively correlated to an increased interaction events between Notch1 and Delta1 (*r* = 0.8943, *P* < 0.003), Notch1 and Delta4 (*r* = 0.8389, *P* < 0.01), Notch1 and Jagged1 (*r* = 0.8743, *P* < 0.005), Notch4 and Delta1 (*r* = 0.9209, *P* < 0.002), Notch4 and Delta4 (*r* = 0.9171, *P* < 0.002), and Notch4 and Jagged1 (*r* = 0.9238, *P* < 0.002), respectively.

## 4. Discussion

The hemodynamic state in the human AVMs appears to be altered [2]. An increasing WSS increases the risk of hemorrhage and induces vascular remodeling in the AVM. However, the mechanism remains a mystery. In this study, we compared the changes of hemodynamic state and Notch signaling activation before and after an arteriovenous fistula creation in rats. We found that an increasing WSS enhances the interaction events between Notch receptors and their ligands, resulting in a significant activation of Notch signaling in “nidus” vessels in the rat AVF model. A mechanism of an increasing WSS associated to AVM formation could be through the activation of Notch signaling pathways in blood vessel endothelial cells.

### 4.1. An Increasing WSS in the AVF Model

The shear stress against the vascular wall of the arterialized vein increased 11-fold over the study period, and shear amplitude linearly correlated with time. In an AVM, the inflow of feeding arteries is pulsatile and blood flow in the nidus is nonuniform, whereas the outflow from draining vein is probably relatively uniform. Vascular walls are elastic, which results in a variable *R*_*i*_. Poiseuille's equation is applied to transform flow rate into average shear stress. Since wall velocity is always zero, increasing flow rate results in a rise of velocity difference between the flow and the wall surface. The shear stress is directly correlated to the above velocity difference. As observed from our experiments, therefore, increasing flow rate is positively correlated to shear stress even if it is not an accurate quantification. Nevertheless, the level of shear stress shown in this study is a general estimation. It is likely that the molecular changes observed in the model are a response to the shear stress from increased blood flow following AVF formation.

A primary determinant of the hemodynamic nature of AVMs is an increasing WSS within the nidus. This is a function of the narrowest cross-sectional area of the fistula, with the most significant increases in resistance occurring in the smallest vessels. The majority of AVMs have as their narrowest point vessels that are greater in diameter than normal capillaries and therefore have a lower resistance than the normal cerebral vasculature [3, 4, 36]. Lower resistance and increased vessel diameter in the nidus lead to increased flow velocity. An important consequence of high flow through the fistula is hypotension in the dilated feeding arteries [3, 4]. This situation is predicted from Poiseuille's equation and has been confirmed in human AVMs by measurement of intra-arterial pressures by direct puncture or superselective catheterization [37]. Reported pressures vary from 45 to 71% of concurrently measured femoral or radial artery pressures [3]. This is significantly lower than normal distal pial arterial pressure, these being around 90% of systemic arterial pressure.

The draining veins appear to be relatively hypertensive compared with normal cerebral veins [4]. Hypertension has been confirmed by pressure measurements in the superficial draining veins at surgery. Deep veins are more difficult to access, although angiographic findings of slower transit of contrast in the deep system relative to the superficial system suggest higher pressures in the deep system [4]. The pathogenesis of spontaneous hemorrhage from AVMs is likely to be multifactorial, involving both anatomical and physiological components. High transmural pressures associated with vascular wall fragility would be expected to produce hemorrhage. It has been reported that the likelihood of presentation with hemorrhage from an AVM was positively correlated with feeding artery pressures [38]. This may explain why larger AVMs, having lower feeding artery pressures, appear to present less frequently with hemorrhage. Higher feeding artery pressures in small AVMs may lead to larger volume hemorrhages due to a higher driving arterial force [4]. An increase in venous resistance due to venous drainage occlusion or stenosis may increase the risk of hemorrhage [3, 4]. Platelet aggregation and thrombosis that occur secondary to turbulence in the nidus and draining veins may compromise the outflow of the lesion and cause hemorrhage.

### 4.2. An Increasing WSS Activates Notch Signaling in the Rat AVF Model

We found that an increasing WSS activates Notch receptors and their ligands in the rat AVF model. The pathogenesis of AVMs was related to the alteration of molecular signaling pathways that regulate vascular homeostasis [39, 40]. The Notch signaling pathway is hypothesized to contribute to AVM pathogenesis via abnormal regulation of vascular development and maintenance. Notch signaling activation was ubiquitous in that activation was observed in both Notch1 and 4 via interaction with their ligands Delta1, Delta4 and Jagged1. The expression of Notch1 and 4, Delta1, Delta4 and Jagged1, and Notch downstream target Hes1 was observed in the endothelial cells of “nidus” vessels in the rat AVF model. The expression of multiple receptors and ligands indicates that activated Notch1 and Notch4 signaling pathways interact between each other. As Hes1 is a downstream target of both activated Notch1 and Notch4 signaling pathways [41], its expression in the endothelial cells of “nidus” vessels in the AVF rats indicates overall activation of Notch signaling in both pathways.

Notch1 and 4 signaling was activated through interaction with Delta1, Delta4, and Jagged1 in the endothelial cells of AVMs. Evidence obtained from loss of function studies supports a critical function of Notch1- and Notch4-mediated signaling in vascular maintenance; disruption of Notch1 results in vascular immaturity and hyperplasia, and even death due to vascular complications [18]. Activation of Notch1 signaling has been reported to result in vessel enlargement and the arteriovenous communication [9, 11]. Notch4 signaling was also activated through interaction with Delta1, Delta4, and Jagged1 in the endothelial cells in the rat AVF model. Previous studies have demonstrated that increasing Notch4 activation in the mouse vasculature produces dilated vessels, reduced smooth muscle cell populations, and arteriovenous communications within the cerebral circulation [10]. Studies also demonstrated the cessation of Notch4 activation stops the progression vascular abnormalities and promotes reversion to the normal vasculature [13]. The observed expression of Notch1 and 4 activation in the endothelial cells of “nidus” vessels in the rat AVF model suggests that an increasing WSS contributes to AVM formation.

The interaction between Notch receptors and their ligand in the vascular development and homeostasis has yet fully characterized. Notch ligands are known to display different binding affinities and suborgan patterns of expression [42]. It would appear that ligand-specific function is dictated by the geographical location of the receptor. Delta1 is expressed in the venous and arterial vasculature during angiogenesis [43]. Inactivation of Delta1 has been observed to impact the overall strength and integrity of the vascular wall [19]. It has been hypothesized that activation of Notch signaling through Delta1 might be associated with the abnormal vascular maturation and arteriovenous specification [25]. Delta4 is expressed throughout the development of both veins and arteries. Delta4 mimics the expression of Notch1 and has been suggested to be the primary activator of Notch1 during angiogenesis [43]. Inactivation of Delta4 has been shown to disrupt remodeling of the vascular plexus with complications in the organization of the vascular bed, vessel diameter, arterial branching, and arteriovenous communication and inhibition of vessel sprouting during angiogenesis [20, 21, 44]. Jagged1 is expressed throughout the development of vasculature [16, 42]. Inactivation of Jagged1 results in insufficient remodeling of the vascular plexus with the loss of vascular integrity and depleted smooth muscle cell population [22, 23]. Jagged1 is involved in insufficient homeostatic maintenance of the tunica media in AVM pathogenesis. Notch activation controls endothelial cell behavior via which receptor-ligand interaction is modified independent of transcriptional regulation, posttranslational modification, and cellular trafficking. Briefly, Notch ligands on the signal-sending cell trigger Notch1 or 4 on the adjacent signal-receiving cell, leading to sequential receptor cleavages within the transmembrane domain, resulting in the release of the Notch intracellular domain (NICD). NICD moves into the signal-receiving cell nucleus and binds to transcriptional factor j kappa-recombination signal-binding protein (RBP-j). Association of NICD and RBP-j replaces the core-pressors with a coactivating complex containing Mastermind-like protein and activates the transcription of target genes such as Hes. The activated Notch signaling downregulates vascular endothelial growth factor (VEGF) receptor2 and upregulates VEGF receptor1, leading to cell differentiation during angiogenesis [17, 45, 46]. In this study, the expression of Notch receptor1 and 4, Jagged1, Delta-like1 and Delta-like4, and Hes1 suggests that upregulation of Notch signaling in response to an increasing WSS is via a “universal” modulator that does not discriminate between ligand or receptor type.

It is generally considered that AVMs are congenital abnormalities that fail to regress [47]; however, by suppressing Notch4 transgene could result in reprograming arterial endothelial cells in the enlarged AVM vessels to a venous endothelial cell specification, leading to a decrease in AVM vessel size [13]. Observation of postnatal AVM formation [48] and the reoccurrence of de novo AVM growth in the adult vasculature after surgical resection [49, 50] support the notion that AVMs are dynamic and proliferative pathologies. This study suggests that WSS could activate reprograming of vascular endothelial cells of AVMs by activation of Notch receptors and their ligands.

A question that pertains to the implication of Notch signaling in the rat AVF model is whether Notch activation is due to angiogenesis or is a secondary effect of endothelium response to an altered state of hemodynamic stress following an AV-fistula formation. VEGF was upregulated in the “nidus” vessels of the rat AVF model over a period of 84 days after creation of an AV-fistula [29], suggesting that angiogenesis occurred. In the current study, examination of “nidus” vessels in the same AVF model revealed that Notch signaling was activated in the endothelial cells of AVM-like vessels. It is possible that an increased hemodynamic stress following an AV-fistula formation induces the activation of Notch signaling, contributing to the angiogenesis of AVM “nidus.”

## 5. Conclusions

An increasing vascular wall shear stress induces apoptosis, and activates Notch1 and 4 signalling pathways in blood vessel endothelial cells, which may contribute to the angiogenesis of AVMs, suggesting a possible mechanism associated with AVM formation and/or reoccurrence of AVMs after surgical resection. This mechanism requires further validation in human.

## Figures and Tables

**Table 1:** Antibodies used in immunohistochemistry.

Antibodies	Dilution	Suppliers
Notch receptor1	1 : 50	R&D System, Minneapolis, MN, USA
Notch receptor4	1 : 50	Cell Signaling, Beverly, MA, USA
Delta-like1	1 : 500	Rockland, Gilbertsville, PA, USA
Delta-like4	1 : 500	Rockland, Gilbertsville, PA, USA
Jagged1	1 : 500	Rockland, Gilbertsville, PA, USA
Hes1	1 : 200	Abcam, Cambridge, UK
Caspase3	1 : 500	Abcam, Cambridge, UK
CD31	1 : 1,000	Abcam, Cambridge, UK
Alexa Fluor 488 goat anti-rabbit IgG	1 : 800	Molecular Probes, Eugene, OR, USA
Alexa Fluor 594 goat anti-mouse IgG	1 : 800	Molecular Probes, Eugene, OR, USA

## Abbreviations

AVF: Arteriovenous fistula
AVMs: Arteriovenous malformations
CCA: Common carotid artery
EJV: External jugular vein
FIU: Fluorescence intensity units
PLA: Proximity ligation assay
WSS: Wall shear stress.
